# Glances at the Hospitals

**Published:** 1902-10-11

**Authors:** 


### GLANCES AT THE HOSPITALS.

### BRISTOL ROYAL INFIRMARY.

The total ordinary income amounted to £11,467, and the total ordinary expenditure to £15,252, showing a deficit of £3,785. Legacies to the amount of £4,724 were received, but in spite of this the year closed with a balance due to the treasurer of £9,321. At the beginning of the year there were 192 in-patients, and 3,057 fresh cases were admitted, making a total of 3,246. The average daily number during the year was 207, and the average residence of each patient was 23 days. Of the total number 2,645 were discharged cured or relieved, 188 died, 17 were dead when taken to the infirmary, and 207 remained at the end of the year. During the year 730 major operations were performed, and an anæsthetic table shows that chloroform was given 823 times, chloroform and ether 45 times, ether 156 times, nitrous oxide and ether 327 times, and nitrous oxide 350 times. An analysis of the more important medical and surgical cases is given. Among other diseases we notice that 59 cases of diphtheria were treated, and of these 53 were cured or relieved and 6 died. There were 19 cases of ulcer of the stomach in the medical wards and all these recovered. On turning to the surgical tables we find that there were 2 cases of ulcer of the stomach, both cured or relieved, and 1 case of perforated ulcer of the stomach, in which death took place. It is here that the tables do not give sufficient information. So, under the heading of tumours, it is stated that there were 49 cancers, all being cured or relieved. To be perfectly satisfactory medical and surgical tables should have separate columns showing age and sex of the patient, duration of treatment, result—"cured," "relieved," or "died"—and a separate column should be left for remarks in special cases. The death-rate was 5·2 per cent. The cost of each bed occupied was £59 13s., and the cost of each in-patient was £3 16s.

### WEST NORFOLK HOSPITAL AT LYNN.

The total ordinary receipts amounted to £2,412, and the total ordinary expenditure to £2,559, and there was a balance of £289 in the treasurer's hands. It is refreshing to meet with a hospital that can keep its expenditure so well within its income, or increase its income to cover its expenditure, and such is the rather uncommon feat of the governors of this charity. On January 1st there were 24 patients in residence, and 496 were admitted subsequently, making a total of 520 in-patients. Of this total 423 were discharged cured, 10 left at their own requests, and 36 died, leaving 49 in the hospital on December 31. The average number of beds occupied was 42, and the average stay of the patients in the wards was 29 days. The cost of each patient was £3 16s. and the cost of each bed occupied was £46 11s. per annum. The cost of food was £15 5s. per bed occupied. Drugs and instruments amounted to £3 2s. per bed, and stimulants to 26s. The average cost of food per head per week for the household and patients was 4s. 5d., and the average daily cost per in-patient was 2s. 6d., which sum included everything. It is much to be desired that every county infirmary would publish these minute and accurate details. They are most useful for purposes of comparison, and they show the governors and subscribers of hospitals those points wherein their own hospital is either careful or extravagant. The medical and surgical tables are equally good. We notice that 22 cases of enteric fever were under treatment, and on following the columns we find that 18 of these recovered, that 3 died, and that one was under treatment on the date of the report. Of gastric ulcer 4 were treated and all recovered. The operation table names all the diseases necessitating the operation, shows the results, and has a separate column for remarks. Chloroform was administered 136 times, and gas and ether 6 times.

### GLASGOW HOSPITAL FOR SICK CHILDREN.

The ordinary income amounted to £4,542, and the ordinary expenditure to £5,194, showing a deficiency in the year's income of £652. The managers say they hope that the subscribers will see to it that in the current and subsequent years the ordinary expenditure is fully met by the ordinary income. We hope so too, but in the meantime it is the duty of the managers to keep the expenditure under their income. At the beginning of the year the hospital contained 72 patients; 738 were admitted during the year, making a total of 810. There were 106 deaths, but of these 31 died within 48 hours of admission. The average daily number under treatment was 64, and the average residence in the hospital was 24 days. The daily cost of each patient was 3s. 4d.; the total cost of each patient discharged was £4 2s. 10d., and the cost of each bed occupied for the year was £61 3s. A table is given which names the various diseases from which the patients were suffering, and the number of patients suffering from each is given, but no results are stated. It is not much use to be informed that there were 33 cases of pneumonia, or 26 cases of empyema, when we are left in ignorance of the duration of the diseases, the treatment employed, and the results. It is the same with the operation table, which shows that 381 operations were performed during the year, but how many of these were successful we do not know. It is true that a table showing the causes of death is printed on pages 19 and 20. We point out these omissions with the hope that next year's report of this most useful institution will have more carefully compiled medical and surgical tables.

